# Anthropometric Parameters as Indicators of Obesity in Adolescents in Montenegro

**Published:** 2018-11

**Authors:** Ivan VASILJEVIC

**Affiliations:** Dept. of Physical Culture, Faculty for Sport and Physical Education, University of Montenegro, Podgorica, Montenegro

## Dear Editor-in-Chief

Obesity today presents a common chronic health problem that lowers quality of life and significantly affects morbidity and overall mortality (1). Global prevention of overweight and obesity in children increased from 4.2% in 1990 to 6.7% in 2010 and is expected to reach 9.1% by 2020 (2). Being obese in childhood is a serious risk factor for the development of obesity in adulthood, which affects the general state of health of an individual, by increasing the risk of developing diabetes, hypertension, coronary arthritis and metabolic syndrome (3).

The aim of the research was to determine the level of obesity in adolescents in Montenegro by the means of several indicators, as well as to compare the results with research conducted in other populations. Level of nutrition was also assessed by other indicators, such as waist-to-height ratio (WHtR) and body fat percentage (body fat %).

The population in this study was made of adolescents aged 14–18 yr with their place of residence on the territory of Montenegro, while the sample of respondents was organized by combining and stratifying, so that various properties and different areas of the mentioned population were processed. The total sample of respondents comprised of 1,449 adolescents from the secondary schools in Montenegro, and the research included all three regions in Montenegro. The people who were taking measures were qualified for this job with anthropometric training before they were allowed to work with research participants. Informed consent was taken from the participants before the study.

The criteria for exclusion from the research were as follows: student refusal, medical conditions such as severe genetic diseases (e.g. Down syndrome or Marfan syndrome), severe hormone disorders, diseases leading to swelling of the sub-cutaneous tissue, diseases leading to loss of muscle mass, metabolic bone diseases and taking drugs that could affect the body mass index.

As indicated in the results of the research, obesity in male adolescents for all regions in Montenegro amounts to 15.2%, while in female adolescents the obesity reaches 9.7%. Analyzing the results of obesity in adolescents in other populations in Europe (4), Slovakia (8%), Russia (9%), Czech Republic (9%), Netherlands (11%), Poland (12%), Germany (13%), Denmark (17%), Bulgaria (17%), Croatia-Zagreb (20%), Great Britain (21%), Spain (21%), Greece-Thessaloniki (22%) and Cyprus (23%), it is clear that obesity in Montenegro also slowly becomes a problem of modern era, especially concerning male adolescents and adolescents living in the northern and central regions in Montenegro. When it comes to adolescents in Poland in Krakow (5) 15.6% of males and 13.4% of females are obese. In Spain (6), obesity in adolescents amounts to 25.6% for boys and 19.1% for girls. In Arab countries (7) obesity among adolescents is a problem that is troubling and disturbing (in Kuwait men 60.4% and women 41.4%), United Arab Emirates - Sharjah city (38.9% men, women 20.2%) and Jordan - Amman (men 31.8%, women 22.1%). The increase in overweight and obesity is also present in school age, and research results show 21% of obesity rate in India in Bangalore (8). When it comes to children in Japan the percentage of obesity is lower and amounts to 5.8% (9), while in South Africa the percentage of obese children amounts to 5.7% (10).

Obesity in adolescents in Montenegro is increasingly present and identifies with other countries in Europe. The percentage of obesity is higher in male population and it is more frequent in the northern and central regions than in the southern, suggesting that beside hypokinesia, which is the primary cause of obesity, reduced and balanced diet is also of great importance.
